# Supplementary material for: Health care seeking behavior for common childhood illnesses in Birendranagar municipality, Surkhet, Nepal: 2018
Source: PLoS One. 2022 Mar 30;17(3):e0264676. doi: 10.1371/journal.pone.0264676 (PMC8967048; doi:10.1371/journal.pone.0264676)
Supplement: S1 Appendix — (DOCX) [file pone.0264676.s001.docx]

**Introduction and consent**

Namaste! My name is Ganga Tiwari. I am studying Masters of Public Health at Institute of Medicine, Maharajgunj Medical Campus, Tribhuvan University, Kathmandu. I am doing my thesis on the topic “**Health care seeking behavior for illness of under- five year children in Birendranagar municipalty, Surkhe**t” which is a requirement for partial fulfillment of MPH degree.

Your house is selected for the collection of information. For this study I will be asking questions related to sociodemographic status, illness status of your under- five children and health seeking behavior. Participation in this study is completely voluntary. You are free to withdraw at any time, without giving any reason. This study is done for the academic purpose and the findings from the study will be beneficial to design the public health program in Surkhet District. Your response is very much valuable for this study. All the information provided by you will be kept confidential and it will be used for academic purpose only. If you have any queries about the study, you can ask freely.

May I proceed with the questions?

Respondent agree to be interviewed

Respondent do not agree to be interviewed

Signature of the respondent:………………………….

Date of the interview:…………………………………….

If respondent do not agree to participate then ending the interview and giving thanks to respondents.

**Interview Schedule**

Factors Associated with Health Care Seeking Behavior on Perceived Illness of Under Five Year Children among Caretakers In Birendranagar Municipality, Surkhet.

Participant Identification Number (ID): …………………………………..

**Section -A**

| **SN.** | **Location and date** | **Response** |
| --- | --- | --- |
| 1 | Ward number / name of ward | ……………………………………… |
| 2 | Date of completion of the interview | …………………………………….. |

**Section –B Demographic Information of the caretakers**

| **SN.** | **Questions** | **Response and Code** |
| --- | --- | --- |
| 1. | How old are you?  (**Age in completed years)** | ……….…………………………………. |
| 2. | Which religion do you follow? | Hindu……………………………….1  Buddhism……………………………2  Christian……………………………3  Islam……..……………………….4  Others……………………………..5 |
| 3. | In which ethnicity do you belong? | Brahmin……………………………1  Chhetri…………………..................2  Aadibasi/Janajati…………………..3  Dalit……………………………….4  Thakuri/Sanyashi………………….5  Muslim…………………………….6  Others………………………………7 |
| 4 | What is your educational qualification? | Literate……………………………1  Illiterate……………………………2 |
| 5 | If literate up to how many grades you have studied?  (completed grades) | ……………………………………. |
| 6 | What is your present occupation? | Home maker………….......................1  Agriculture………………………….2  Service………………………………3  Business…………………………….4  Labor…………..................................5  Others………………………..6 |

| **7.** | What is the present occupation of head of the family? | ……………………………………. |
| --- | --- | --- |
| **8.** | What is the educational qualification of head of the family? | ……………………………………. |
| **9.** | What is your average monthly family income?  **(Income in Nepalese Rupees per month)** | ……………………………………. |
| **10.** | What is your marital status? | Married………………………….1  Divorced………………………...2  Separated………………………..3  Widowed………………………..4  Unmarried ……………………...5 |
| 11. | In which type of family do you belong? | Nuclear family…………………..1  Joint family…...............................2 |
| 12. | How much time does it take to reach the nearest health facility?  **(time in minute)** | …………………………………… |

**Section C**

**Questions related to health care seeking behavior**

| 13 | How many children do you have? | …………………………………… |
| --- | --- | --- |
| 14 | What is your relationship with child? | Mother ……………………………….1  Father ………………………………..2  Grandmother………………………....3  Grandfather…………………………..4  Others………………………………..5 |
| 15 | Sex of the child? | Male…………………………………1  Female……………………………….2 |
| 16 | Where was your child born? | Health facility………………………..1  Home…………………………………2 |
| 17 | Birth order of the child? | First…………………………..............1  Second ……………………………….2  Third …………………………………3  Fourth and higher…………………….4 |
| 18 | Age of the child in months?  Could you please bring child’s birth certificate, child immunization card, and any immunization record from a private health provider? | ……………………………………….. |
| 19 | Did your child suffer from diarrhea in last one month duration? | Yes…………………………………...1  No………………………………….....2 |
| 20 | Did your child have cough in last one month duration? | Yes…………………………………...1  No……………………………….........2 |
| 21 | Did your child have difficulty breathing in last one month duration? | Yes…………………………………...1  No……………………………………2 |
| 22 | Did your child have fever in last one month duration? | Yes…………………………………..1  No……………………………………2 |
| 23 | How many number of symptoms did your child suffered in last one month duration? | Only one …………………………….1  Two…………………………………..2  Three…………………………………3  More than three ……………………...4 |
| 24 | For how many days your child became ill? | ………………………………….......... |
| 25 | What did you perceive about severity of the illness? | Illness was mild……………………...1  Illness was moderate…………………2  Illness was severe…………………….3 |
| 26 | Did you seek any treatment or care from any source for your child during illness? | Yes…………………………………...1  No…………………………………....2 |
| 27 | What treatments or care did you seek? | Home remedies/self- treatment………..1  Purchased medicine from pharmacy/drug shops…………………………………..2  Went to health facility………………...3  Went to traditional /spiritual healers…..4  Sought advice from FCHVs…………...5  Others specify……………………….....6 |
| 28 | What treatment or care did you seek at first? | Home remedies/self- treatment………..1  Purchased medicine from pharmacy/drug shops…………………………………..2  Went to health facility………………...3  Went to traditional /spiritual healers…..4  Sought advice from FCHVs…………...5  Others specify……………………….....6 |
| 29 | If used home remedies then what home remedies or self- treatment used? | ……………………………………………………………………………………………………………………………………………………………………………………………… |
| 30 | If home remedies then, what were the reason for using home remedies? | ……………………………………………………………………………………………………………………………………………………………………………………………… |
| 31 | If traditional healer, then what were the reasons for consulting traditional healer? | ………………………………………..……………………………………………………………………………………………………………………………………………………… |
| 32 | If health facility, then what were the reasons for consulting health facility? | …………………………………………………………………………………………………………………………………………………………………………………………………………………………….. |
| 33 | What was the condition of child illness after first time care seeking? | Improved……………………………..1  Not improved……………………………..2 |
| 34 | Did you consult second care provider after consulting first provider for the treatment of your child illness? | Yes……………………………….......1  No……………………………………2 |
| 35 | From which provider did you shift? | Drug seller/pharmacist to health facility…………………………….....1  Government health facility to private health facility………………..........................2  Private to government health facility………………………………..3  Traditional healers to health facility….............................................4  Health facility to traditional healer…………………………………5  Other specify………………………….6 |
| 36 | Why did you switch to second provider? | ……………………………………….  ………………………………………..  ……………………………………….. |
| 37 | What were the reasons for not seeking any treatment or care for your child? | Far distance to reach health facility.....1  Illness was mild.................................2  Busy work schedule............................ 3  Waited for self- recovery.………........4  Treatment was expensive.....................5  Unfriendly behavior of the health professional…………………………..6  Long waiting hour to visit doctor…….7  Other specify.………………………....8 |
| 38 | Who decides to take the sick child for treatment in your family? | Mother herself ……………………….1  Father………………………………...2  Grandparents ………………………...3  Other family member……..…………4 |

**Section-D**

**Knowledge of caretakers on danger signs of under- five illnesses**

| 39 | Did you have heard/know about general danger signs among under- five children? | Yes…………………………………………...1  No………………………….............................2 |
| --- | --- | --- |
| 40 | If yes, then from which source did you know? | Radio…………………………………………1  Television…………………………………….2  Newspaper……………………………………3  Internet……………………………………….4  FCHVs/Health workers……………………...5  Friends/Relative/neighbor…...........................6  Others……………………………………….... |
| 41 | When should a sick under -five child be taken immediately to the health facility? | Child not able to breastfeed…………………1  Child develops fever………............................2  Child becomes sicker………...........................3  Child drinks poorly….……............................4  Child has fast breathing……………………...5  Child has difficulty in breathing……………………………………..6  Child has blood in stool……………………...7  Child becomes lethargic or unconscious………………………………….8  Child vomits everything…………………….9  Child develops convulsion..............................10  Others……………………...............................  ………………………………………………..11 |

**Section -E**

**Health system related factors**

| 42 | Did you visit health facility for the treatment of your child? | Yes ……………………………...1  No……………………………….2 |
| --- | --- | --- |
| 43 | After how many days did you visited health facility? | ………………………………  …………………………….... |
| 44 | Which type of health facility did you preferred to treat your child? | Government…………………….1  Private…………………………..2 |
| 45 | What were the reasons for selecting government health facility? | ……………………………....  …………………..…………..  ……………………………….  ……………………………… |
| 46 | What were the reasons for selecting private health facility? | ………………………………  ………………………………  ……………………………… |
| 47 | How much waiting time does it usually take to consult a health professional in health facility? | …………….……………… |
| 48 | How would you describe quality of care you received from health facility? | Very good……………………….1  Good…………………………….2  Fair………....................................3  Not so good………...……...........4 |

**Thank you very much for your participation!**
