# Supplementary material for: Health care seeking behavior for common childhood illnesses in Birendranagar municipality, Surkhet, Nepal: 2018
Source: PLoS One. 2022 Mar 30;17(3):e0264676. doi: 10.1371/journal.pone.0264676 (PMC8967048; doi:10.1371/journal.pone.0264676)
Supplement: S2 Appendix — (DOCX) [file pone.0264676.s002.docx]

**परिचय तथा मंजुरिनामा**

नमस्कार !

मेरो नाम गंगा तिवारी हो । म महाराजगंज मेडिकल क्याम्पस ,चिकित्सा शास्त्र संस्थान काठमाडौँमा जनस्वाथ्य विषयमा स्नाकोत्तर दोस्रो वर्षमा अध्यन गर्दै छु। यस तहको अध्ययनको शिलशिलामा म " **विरेन्द्रनगर नगरपालिकामा पाँच वर्षभन्दा मुनिका बालबालिकाहरू बिरामी हुँदा , आभिभावकहरुले गर्ने स्वास्थ्य सेवा सम्बन्धि खोजी र यसका विभिन्न तत्वहरु**” नामक शिर्षक मा अध्ययन गर्दै छु। यस अध्ययनकालागी मैले , समाजिक ,जनसाङ्खिक,आर्थिक स्थिति तथा स्वास्थ्य सेवा सम्बन्धि विभिन्न प्रश्नहरु सोध्नू पर्ने हुन्छ।

तपाइको घर यस अध्ययनमा जानकारी लिनको लागि चयन भएको छ। तपाइले दिएको जानकारी शे}क्ष्यिक उधेश्यकोलागि मात्र प्रयोग गरिनेछ। यस अन्तरबार्ताका लागि करिब २० मिनेट समय लाग्ने छ। तपाइको नाम कहिँपनि उल्लेख गरिने छैन । तपाइले दिएको जानकारी र यस अध्यनको निष्कर्षबाट सुर्खेत जिल्लामा बाल स्वास्थ्य सम्बन्धि निति तथा कार्यक्रमहरु निर्माण गर्न सहयोग मिल्नेछ। तपाइको प्रतिकृया यस अध्यनका लागि एकदमै महत्वपुर्ण हुनेछ। तपाइले दिएका जानकारीहरु गोप्य राखिनेछन्। यस अध्ययनका कारणले तपाइलाई कुनै पनि किसिमको नकरात्मक असर नपर्ने विश्वास दिलाउन चाहन्छु।

यदि तपाइलाई अध्ययन सम्बन्धी कुनै जिज्ञासा भएमा निर्धक्क सोध्न सक्नू हुनेछ।

यदि तपाई यस अध्ययन मा सहभागी हुन मंजुर हुन्हुन्छ वा हुनुहुन्न , कृपया तलको खाली कोठामा(√) गरी हस्ताक्षर गर्नु होस्। यदि मंजुर हुनुहुन्छ भने तलका प्रश्नहरुको उत्तर दिनुहोस्।

सहभागिता को लागी मन्जुर छु

सहभागिता को लागी मन्जुर छैन

सहभागीको सहि…………………………………

मिति :…………………………………………….

सहभागी सहमत नभएमा - धन्यवाद दिदै अन्तर्वार्ता अन्त्य गर्ने .

**अन्तरबार्ता तालिका**

**सहभागी परिचय नम्बर…………**

**भाग- क**

| **क्र. श** | **स्थान र समय** | **प्रतिकृया** | **कोड** |
| --- | --- | --- | --- |
| १ | वडा नम्बर | …………………………………… |  |
| २ | अन्तरबार्ता मिति | …………………………………………. |  |

**भाग –ख**

**सामाजिक जनसंखिक विवरण**

| **क्र. श** | **प्रश्न** | **प्रतिकृया** | **कोड** |
| --- | --- | --- | --- |
| १ | तपाई कति वर्ष पुग्नुभयो ?  **(उमेर पूरा वर्षमा)** | ………………….. |  |
| २ | तपाई कुन् धर्म मान्नु हुन्छ ? | हिन्दु…….. १  बौद्ध …..२  क्रिस्चियन….३  मुस्लिम………४  अन्य……………५ |  |
| ३ | तपाई कुन समुदाय अन्तर्गत पर्नु हुन्छ? | ब्राह्मण ………………………१  क्षेत्री……………………………..२  आदिवासी / जनजाती………३  दलित……………………………….४  &s'/L/:fGof;L……………………5  d'l:nd………………………..^  अन्य………………………………& |  |
| ४ | तपाईको मुख्य पेशा के हो ? | गृहिणी……….१  कृषि……………२  सेवा…………….३  व्यापार…………४  मजदुर………….५  अन्य…………….६ |  |
| ५ | तपाईको शे}क्ष्यिक योग्यता के हो? | साक्षर…………१  निराक्षर…….. २ |  |
| ६ | यदि साक्षर भए कति कक्ष्यासम्म पढ्नु भएको छ ?  (पास भएको कक्ष्या) | ………………………………………………………. |  |
| ७ | तपाईको घरको घरमुलीको शैक्षिक योग्यता कति हो ? | ……………………………………………………….. |  |
| ८ | तपाईको घरको घरमुलीको मुख्य पेशा के हो ? | ………………………………………………………. |  |
| ९ | तपाईको घरको मासिक आम्दानी कति हुन्छ?  **(आम्दानी नेपाली रुपैंयामा)** | ………………………………………………………. |  |
| १० | तपाईको बैवाहिक सम्बन्ध के हो ? | विवाहित…………………………….१  सम्बन्ध बिक्ष्यद्…………………२  विदुवा/ विदुर …………………….३  विभाजित……………………………४ |  |
| ११ | तपाईको कस्तो किसिमको परिवारमा बस्नु हुन्छ ? | एकल……………१  संयुक्त…………..२ |  |
| १२ | तपाईलाई नजिकैको स्वास्थ्य संस्था जान कति समय लाग्छ ? | ………………………………………………………… |  |

**भाग –ग**

**स्वास्थ्य सेवा खोजी सम्बन्धि प्रश्नहरु**

| १३ | तपाईको घरमा कति वटा बच्चाहरु छन् ? | ……………………................................... |  |
| --- | --- | --- | --- |
| १४ | तपाईको बच्चासंग को नाता के हो ? | आमा……………१  बुवा………………२  हजुरआमा……….३  हजुरबुवा………..४  अन्य……………..५ |  |
| १५ | तपाईको बच्चाको उमेर कति भयो ?  (जन्म दर्ता अथवा खोप कार्ड छ भने , कृपया एकछिनको लागि देखाउनु होस्,**उमेर महिनामा)** | …………………………………………………………………….. |  |
| १६ | तपाईको बच्चाको लिङ्ग के हो ? | छोरा………………१  छोरी………………२ |  |
| १७ | तपाईको बच्चा कहाँ जन्मिएको थियो ? | अस्पतालमा ………..१  घरमा ………………….२ |  |
| १८ | तपाईको बच्चाको जन्म अनुक्रम कुन हो ? | पहिलो…………१  दोश्रो…………...२  तेश्रो…………….३  चौंथो वा सो भन्दा बढि…….४ |  |
| १९ | के तपाईको बच्चालाई गत एक महिनामा पखाला लागेको थियो ? | थियो……………….१  थिएन…………….२ |  |
| २० | के तपाईको बच्चालाई गत एक महिनामा खोकी लागेको थियो ? | थियो……………….१  थिएन…………….२ |  |
| २१ | के तपाईको बच्चालाई गत एक महिनामा सास फेर्न अफ्ठेरो भएको / कोखा हानेको थियो ? | थियो……………….१  थिएन…………….२ |  |
| २२ | के तपाईको बच्चालाई गत एक महिनामा ज्वरो आएको थियो ? | थियो……………….१  थिएन…………….२ |  |
| २३ | तपाईको बच्चालाई गत एक महिनामा भित्रमा कति वोटा लक्ष्यण देखा पर्यो ? | एक………………….१  दुई…………………..२  तिन………………….३  चार वा सो भन्दा बढि…….४ |  |
| २४ | तपाईको बच्चा कति दिनसम्म बिरामी पर्यो ? | ……………………………………………………………………. |  |
| २५ | तपाइको बच्चा कत्तिको बिरामी पर्यो ? | सामान्य………..१ मध्यम …….२ सारै/सिकिस्त …………………….३ |  |
| २६ | बच्चा बिरामी हुँदा तपाईले के गर्नु भयो ? **(यदि उपचार नगरेको भए प्रश्न न. ३८ )** | उपचार गरे……………..  उपचार गरेन……………. |  |
| २७ | तपाइले आफ्नो बच्चा बिरामी हुदा के -के उपचार गर्नु भयो ? | घरेलु उपचार ………………………. १  औषधि पसलमा जाचँ ………….२  स्वास्थ्य संस्थामा जाचँ ……….३  झारफुक.................................... ४  आयुर्वेदिक स्वास्थ्य संस्थामा जाचँ ….. ५  स्वास्थ्य स्वयमसेविकासंग परामर्श …..६  अन्य ………………………………………७ |  |
| २८ | यदि उपचार गर्नु भएको थियो भने, पहिला के उपचार गर्नु भयो ? | घरेलु उपचार ………………………. १  औषधि पसलमा जाचँ ………….२  स्वास्थ्य संस्थामा जाचँ ……….३  झारफुक.................................... ४  आयुर्वेदिक स्वास्थ्य संस्थामा जाचँ ….. ५ स्वास्थ्य स्वयमसेविकासंग परामर्श …….६  अन्य ………………………………………………………७ |  |
| २९ | यदि घरेलु उपचार गर्नुभएको भए, के-के प्रयोग गर्नु भयो? | …………………………………………………………………. |  |
| ३० | यदि घरेलु उपचार गरेको/ आफैले औषधि खुवाउनु भएको भए, कारणहरु? | ………………………………………………………………… |  |
| ३१ | यदि झारफुक् गर्नुभएको भए, कारणहरु? | ……………………………………………………………………. |  |
| ३२ | यदि स्वास्थ्य संस्था लैजानु भएको भए, कारणहरु? | ……………………………………………………………….. |  |
| ३३ | पहिलो पल्ट उपचार गरेपछि बच्चाको बिरामी अवस्था कस्तो भयो ? | निको भयो…………… १  निको भएन………….. २ |  |
| ३४ | के तपाई बच्चाको उपचारको लागि पहिलो पछि दोश्रो सेवा प्रदायककोमा जानु भयो ? | गएँ……………..... १  गएन………………. २ |  |
| ३५ | यदि जानु भएको भए,कुन् बाट कुनमा जानु भयो ? | पहिला औषधि पसल तेस्पछि स्वास्थ्य संस्था……..१  पहिला सरकारी स्वास्थ्य संस्था तेस्पछि निजी स्वास्थ्य संस्था……..२  पहिला घरेलु उपचार तेस्पछि स्वास्थ्य संस्था……..३  पहिला झारफुक तेस्पछि स्वास्थ्य संस्था…४  पहिला स्वास्थ्य संस्था तेस्पछि झारफुक…५  अन्य……………………………………………………………………६ |  |
| ३६ | के कारणले गर्दा तपाई दोस्रो सेवा प्रदायककोमा जानु भयो ? | ………………………………………………………………… |  |
| ३७ | तपाईले बच्चाको उपचार नगर्नुका कारणहरु के-के हुन् ? | स्वास्थ्य संस्था टाढा भएकाले…….१  सामान्य बिरामी भएकाले……………२  काममा बेस्त भएकाले………….३  बिरामी आफै निको हुन्छ भनेर …..४  महँगो उपचार भएकाले…………………………..५  स्वास्थ्यकर्मीको ब्यबाहार राम्रो नभएकाले…६  अस्पतालमा लामो लाइन लाग्नू पर्ने भएकाले………………………………………….७  अन्य……………………………………………….९ |  |
| ३८ | तपाईको घरमा बच्चा विरामी हुदा उपचारको निर्णय कस्ले गर्नु हुन्छ? | आमाआफैले…………………………………….१  बाबाले …………………………………………२  हजुरआमाले ……………………………………………३  घरका अन्य सदस्यले…………………….४ |  |

**भाग-घ**

**बच्चामा खतराका चिन्ह सम्बन्धि प्रश्नहरु**

| ३९ | के तपाईले बच्चामा सामान्यतया देखिने खतराको चिन्हहरुको बारेमा थाहा पाउनु/ सुन्नु भएको छ ? | छ……………..१  छैन……………२ |
| --- | --- | --- |
| ४० | यदि थाहा पाउनु / सुन्नु भएको छ भने , कहाँबाट सुन्नु भयो ? | रेडियो………….१  टेलिभिजन………२  पत्रिका…………….३  इन्टरनेट………….४  स्वास्थ्य स्वयेम्सेबिका/ स्वास्थ्यकर्मी…….५  साथी/ छिमेकी / आफन्त…..६  अन्य………………..७ |
| ४१ | बच्चालाई कस्तो अवस्थामा तुरुन्त स्वास्थ्य संस्थामा लैजानु पर्छ ? | बच्चाले स्तनपान नगरेमा……१  बच्चालाई ज्वारो आएमा………२  बच्चा झन् बिरामी भएमा……३  बच्चाले पिउन नसक]मा……….४  बच्चाले छिटो छिटो सास फेरेमा…………………………………५  बच्चा लाई सास फेर्न गाह्रो भएमा…………………………………६  बच्चाको दिसामा रगत देlखएमा………………………………७  बच्चा सुस्त वा बेहोस भएमा………………………………….८  बच्चाले खाएको जlत सबे} बान्ता गरेमा……………………….९  बच्चालाई कम्पन आएमा…….१०  अन्य………………………………….११ |

**भाग -ङ**

**स्वास्थ्य सेवा सम्बधि प्रश्नहरु**

| ४२ | के तपाईले आफ्नो बच्चालाई स्वास्थ्य संस्थामा/ औषधि पसल उपचार गराउनु भएको थियो ? | थिए……………१  थिएन …………२ |  |
| --- | --- | --- | --- |
| ४३ | तपाइले बच्चा बिरामी भएको कति समयपछि औषधि पसल/ स्वास्थ्य संस्था लैजानु भयो? | ………………………………………………………………. |  |
| ४४ | तपाईले आफ्नो बच्चाको उपचार कुन स्वास्थ्य संस्थामा गराउनु भयो? | सरकारी………….१  निजी……………..२ |  |
| ४५ | तपाईले सरकारी स्वास्थ्य संस्था रोज्नुका कारणहरु के- के हुन्? | …………………………………………………………….. |  |
| ४६ | तपाइले निजी स्वास्थ्य संस्था रोज्नुका कारणहरु के- के हुन् ? | …………………………………………………………………… |  |
| ४७ | तपाईको बच्चालाई स्वस्थ्यकर्मीबाट उपचार गराउन प्राय कति समय प्रतिक्ष्या गर्नुपर्ने हुन्छ? | ………………………………………………………………. |  |
| ४८ | स्वास्थ्य संस्थाबाट पाइने सेवाको गुणस्तरलाई कसरी लिनु भएको छ ? | धेरै राम्रो……………१  राम्रो…………………२  ठिकै…………………३  नराम्रो……………….४ |  |

**तपाइको सहभागिताको लागि धन्यबाद !**
