# Supplementary material for: Health care seeking behavior for common childhood illnesses in Birendranagar municipality, Surkhet, Nepal: 2018
Source: PLoS One. 2022 Mar 30;17(3):e0264676. doi: 10.1371/journal.pone.0264676 (PMC8967048; doi:10.1371/journal.pone.0264676)
Supplement: Attachment — Submitted filename: Response to Reviewer.docx [file pone.0264676.s004.docx]

# Response to the reviewer and editor

Date: October 5, 2020

To the Editor,

PLOS ONE Journal

Greetings! Hope you are fine and doing well. First of all, I would like to thank both the editor and reviewers for the extensive review of our manuscript and providing us opportunities to revise it. I have tried to incorporate all the feedback provided to us. For the revision of this manuscript, I took the support of my colleagues Dr. Sushil Pokhrel and Dr. Ganesh Tiwari. I checked language, spellings, and grammar using software/app for made for checking grammar. I have manually checked each sentence of this manuscript. I have mentioned below a table that explains my response in each comment of the reviewers and editor. Now, I am submitting the revised version of the manuscript as well as the original version with track changes. I am also submitting the supporting information such as tools of data collection in English as well as Nepali language and SPSS data set. Thank you very much for your time and consideration.

Best regards!

Ganga Tiwari

| **SN.** | **Comments by editor and reviewer** | **Response** |
| --- | --- | --- |
|  | **Editor Comments** |  |
| 1 | Table 6, you should list the number of samples for each variable category | Yes, I have listed the number of samples for each variable |
| 2 | Table 6, the definition of Ethnicity is not explained | I have provided an operational definition of ethnicity. |
| 3 | Table 6, is that secondary education includes higher secondary within the education level? | No secondary education includes lower secondary education (6^th^ standard to 10^th^ standard) in our study.  Higher secondary is considered a college education in our study. |
| 4 | Why does Table 6 list the service industry separately? The service industry only accounts for 7%. | I have checked the association of healthcare-seeking behavior with all the occupations categorizing into two group like home makers and non-homemakers/ business and non-business/agriculture and non-agriculture and service and non-service, I did not found any association among other categories but I found association with service and non -service category, so I have listed service and non-service category in table 6. |
| 5 | In table 6, I don't see the definition of socioeconomic status classification. | Now, I have mentioned the definition of socioeconomic class in the operational definition. |
| 6 | Does the distance between Table 6 and health care facilities refer to the geographical distance or the travel time as in Table 2? | Yes, the distance mentioned in table 6 refers to the geographical distance which is measured in travel time as in table 2. But in Bivariate and multivariate analysis I have not categorized distance into below 30 min and above 30 min, as the distance is a continuous variable I directly check the association of distance and healthcare-seeking behavior. It was measured in travel time to reach the nearest health facility in minutes. |
| 7. | Table 6, about the history of difficult breathing, the definition of difficulty? | Now, I have mentioned about the definition of difficulty in breathing in the operational definition. |
| 8. | What is the operational definition of knowledge for dangerous signals in Table 6? | Now, I have mentioned the definition of knowledge of danger signs in table 6 at the operational definition section. |
| 9 | From, Table 2 we know that the reasons for choosing public and private medical institutions are different, and the regression analysis of seeking medical behaviors can be considered as homemakers analysis. | Yes, the regression analysis of seeking medical behavior can be considered as a stratified analysis. |
| 10 | It is recommended that the coding method of the dependent variable be presented below Table 6 for an explanation. | Now, I have mentioned in the data analysis section under methodology. |
| 11 | Is it confirmed that there are no multicollinearity issues? | Yes, I have calculated the Variance Inflation Factor (VIF) among the independent variables selected in the multivariate logistic analysis model. VIF value was less than 2 for every variable checked. So it is confirmed that there is no issue of multicollinearity. |
| 12 | The quality of the logistic regression model and the result of the Hosmer-Lemeshow Test? (less...) | Hosmer Lemeshow test, the goodness of fit of the model was assessed. The test statistic was 0.500 (>0.05) that showed that the model adequately fits the data. |
|  | The authors refer to several studies in the Discussion chapter, sometimes to one or two, other times to other one or two, etc, but no general overview of these studies has been given. Probably the whole paper could be made shorter if a summary (maybe even in a table format) would be presented in the Introduction, and the major points would be stated. Later, then, they only have to refer to this section. | Now, I have tried to make the discussion section shorter by reducing the repeated contents of result and mentioned only major issues. |
|  | Methods are poorly described, especially regarding the interviews. No detailed information is given about the questions of the interviews. At least some typical examples and variations should be presented (maybe as an Appendix). The interview method is typically a crucial information since it can manipulate the interviewees and can lead to false interpretation. It isn’t clear, too, what method for coding had been used. | Now, I have explained the interview technique in the methodology part.  Regarding the coding of the dependent variable, I have now explained in the operational definition. |
|  | A technical note: No explanation is given on the first mention for abbreviation ARI (Page 4), that of FCHV (Page 5), IMCI (Page 6) etc. Although there is a list of abbreviations at the end of the manuscript, the reader does not find it while reading the text. Either the table should be placed onto the beginning of the paper, or abbreviations should be explained when first mentioned. Overall opinion: It is an important topic, but the manuscript requires general improvement (major revision) before accepting. | Now I have explained abbreviations when used at first and then I have kept list of abbreviations at the last section of manuscript. |
|  | **Journal requirement** |  |
|  | Please ensure that your manuscript meets PLOS ONE's style requirements, including those for file naming | Now I have revised my manuscript using PLOS ONE’s style requirement, and I have named file name accordingly. |
|  | We suggest you thoroughly copyedit your manuscript for language usage, spelling, and grammar. If you do not know anyone who can help you do this, you may wish to consider employing a professional scientific editing service | Yes, I have thoroughly copyedited my manuscript for language, spelling, and grammar through grammar checking websites/apps as well as with the help of my colleagues. |
|  | It seems unusual to use 0.2 p-values for statistical analysis; at least some explanation should be given. Discussion is too long, generally because results are repeated several times in this section. Generally, Discussion is an overview of the results obtained is a short and compact format, to give the reader an impression about the meaning of the research. The many references to other studies make it even more complicated (see above). | It has been shown in a study that use of p-value < 0.05 is too stringent and often excludes important variables from the logistic regression model, so choosing a p-value ranging from 0.15 to 0.20 is highly recommended, various published studies have used p-value 0.2 for selecting the variables for multivariate logistic regression. Now I have explained this in the manuscript also.  Now I have tried to make the discussion section shorter by reducing the repeated contents of result and mentioned only major issues |
|  |  |  |
