# Supplementary material for: Health care seeking behavior for common childhood illnesses in Birendranagar municipality, Surkhet, Nepal: 2018
Source: PLoS One. 2022 Mar 30;17(3):e0264676. doi: 10.1371/journal.pone.0264676 (PMC8967048; doi:10.1371/journal.pone.0264676)
Supplement: Attachment — Submitted filename: Response to reviewers.docx [file pone.0264676.s005.docx]

# Response to the reviewer and editor

Date: March 5, 2020

To the Editor,

PLOS ONE Journal

Greetings! Hope you are fine and doing well. First of all, I would like to thank both the editor and reviewers for the extensive review of our manuscript and providing us opportunities to revise it.For the revision of this manuscript, I took the support of my colleagues Dr. Sushil Pokhrel and Dr. Ganesh Tiwari. We have tried to incorporate all the feedback provided in the revised version of the manuscript. I have mentioned below a table that explains my response in each comment of the reviewers and editor. Now, I am submitting the revised version of the manuscript as well as the original version with track changes. Thank you very much for your time and consideration.

Best regards!

Ganga Tiwari

| **Reviewers Feedback** | **Author Response** |
| --- | --- |
| Reviewer #3: **Title**: needs to be modified, can be made short | Yes, the title has been modified and made short |
| **Abstract**: Objective needs to be more specific. Conclusion needs language editing, can be made short. Last statement not a part of study objective | Now, objectives are written more specifically, conclusion section has been edited. |
| Keywords: can be written as per MeSH terms | Now, Key words have been written as per MeSH terms in the revised version of manuscript. |
| Objectives: need to be more specific | Now, the objectives are written more specifically. |
| Methods: section needs to be rewritten. It is very lengthy, can be made short with only relevant details. Sample size and technique can be explained by a flowchart; will make it concise and reflect better understanding. “FCHVs” needs to be spelt out for the first time in text, it can later be used as an abbreviation. Was Kuppuswami scale modified Nepali version validated, before using in study. “Perceived Illness” used in study can be elaborated in operational definition section. Inclusion criteria mentions about only the treatment seeking period of last one month, which is a major limitation. Childhood/under-five manifestations may not always be present, a longer duration would be better like 3 months | We have rewritten methodology section. We have tried to make it short. Sampling technique is now explained by a flowchart. FCHVs are now written in full word when it is first used.  Kuppuswamy scale modified in context of Nepal was used; this version is already validated and used by various published studies in Nepal.  Perceived illness is now defined on the operational definition section.  Illness period of only one month duration was taken to avoid recall bias among the caretakers. |
| Results: require language editing. “Perceived Illness” needs to be explained in methodology. Similarly, “Perceived Severity” mentioned not explained in text. Too many[Table 1 to 5] tables. Can be combined with statistical analysis part of bivariable analysis. Multivariable analysis[Table 6] is fine | Language editing done in result section.  Perceived illness and severity has been defined in the operational definition section. Some tables have been removed. |
| Discussion: is very elaborate; can be shortened in length and only relevant findings highlighted | Discussion section has been made short. |
| Limitations: not mentioned | Now, limitation has been mentioned. |
| References: Some old references may be removed. Recent studies within last 5 years can be cited | Some old references have been removed. |
