# Supplementary material for: Health care seeking behavior for common childhood illnesses in Birendranagar municipality, Surkhet, Nepal: 2018
Source: PLoS One. 2022 Mar 30;17(3):e0264676. doi: 10.1371/journal.pone.0264676 (PMC8967048; doi:10.1371/journal.pone.0264676)
Supplement: Attachment — Submitted filename: Response to the reviewers.docx [file pone.0264676.s006.docx]

# Response to the reviewer and editor

Date: July 31, 2020

To the Editor,

PLOS ONE Journal

Greetings! Hope you are fine and doing well. First of all, I would like to thank both the editor and reviewers for the extensive review of our manuscript and providing us opportunities to revise it. For the revision of this manuscript, I took the support of my colleagues Dr. Sushil Pokhrel and Dr. Ganesh Tiwari. We have tried to incorporate all the feedback provided in the revised version of the manuscript. I have mentioned my response in comment of the reviewers and editor. Now, I am submitting the revised version of the manuscript as well as the original version with track changes. Thank you very much for your time and consideration.

Best regards!

Ganga Tiwari

**Journal Requirements:**

Please review your reference list to ensure that it is complete and correct. If you have cited papers that have been retracted, please include the rationale for doing so in the manuscript text, or remove these references and replace them with relevant current references. Any changes to the reference list should be mentioned in the rebuttal letter that accompanies your revised manuscript. If you need to cite a retracted article, indicate the article’s retracted status in the References list and also include a citation and full reference for the retraction notice.

**Author’s response**: I have reviewed the reference list. It is complete and do not include the retracted article,

**Reviewer's Responses to Questions**

1. The title should be changed to “Factors associated with healthcare seeking behavior for common illnesses amongst under-five children in Surkhet district, Nepal: 2018“

**Author’s response**: I have changed the title accordingly.

2. The manuscript needs to be numbered in order to reference to line numbers. This makes review easier.
Abstract
Introduction of abstract
3. “There are few studies on health care seeking behavior among caretakers in Nepal, so the objective of this study was to itsassess the healthcare-seeking behavior of the caretakers in Birendranagar municipality of Surkhet district. So, this study aims to identify prevailing health care seeking behavior of caretakers on perceived illness of under five year children and to identify the association of socio demographic, economic, illness related and health system related factors with health care seeking behavior.” Should be changed to “There are few studies on healthcare seeking behavior among caretakers in Nepal. Therefore, we conducted this study to determine the level of healthcare seeking behavior of caretakers on perceived illness and to identify the factors associated with health care seeking behavior of the caretakers of under five-year children from Surkhet district in Nepal in 2018.”

**Author’s response:** I have made slight change in the language of the objective and changed the objective, we did not determine the level, and we only determine the different health care seeking behaviors among the care takers.

**Methods section of abstract**
4. Add level of P-value for statistical significance

**Author’s response:** Previously, I had added level of p- value for statistical significance in abstract but previous reviewers of this journal suggested me to remove it from abstract so I have removed it.

5. How the logistic regression models were built (optional depending on word count)

**Author’s response**: Abstract become lengthy, if we add this detail

Results
6. Change “Regarding healthcare-seeking behavior of caretakers for their children’s illnesses, the most common source of care-seeking was visiting pharmacy directly at first (42.4 %), only one quarter (25.3 %) of the caretakers visited health facilities, among those who visited health facilities, only (37.2 %) of caretakers sought prompt health care.” to “Of these, 42.4% visited the pharmacy directly, 25.3% visited the health facilities and XXXX did nothing. Amongst those who visited a health facility, 37.2% of caretakers sought prompt health care.”

**Author’s response:** Thank you for your feedback; I have changed the result section accordingly.

Conclusion
7. Rather than just repeat the results consider using statements like (“There is a need to understand and address individual and socio-economic barriers to health seeking to increase access and use of health care and fast-track progress towards Universal Health Coverage amongst children from Surkhet district in Nepal.” This is what would be an appropriate conclusion that answers the question: How does this study relate to the SDGs and universal health coverage as well as Nepal National Health Strategy?

**Author’s response:** Since, this study is confined to Birendranagar Municipality of Surkhet district, we have concluded according to our study results at the municipality level. Now, we have also included policy implication of our study.

Main body of the paper
8. Include a running title of the paper
Introduction
9. Change the objectives as stipulated in the abstract
Methods
10. p-value should be changed to P-value
11. The authors should make it clear how they arrived at the final model. Did they use log likelihood ratio methods or some other methods? They also need to be clear on what multivariate analysis that they are conducting.

**Author’s response**:

We used SPSS for the data analysis. The twelve variables (ethnicity, educational status, occupation, socioeconomic status, distance to reach nearest health facility, place of delivery, perceived severity, history of difficulty breathing, knowledge on danger signs, number of symptoms, duration of illness, number of children) that exhibited significant association with health care seeking in bivariate analysis i.e. p value ≤ 0.2 were further analyzed in multivariate analysis using binomial logistic regression. Multivariate analysis was done for adjustment of possible confounders. Hosmer Lemeshow test, the goodness of fit of the model was assessed. The test statistic was 0.500 (>0.05) that showed that the model adequately fits the data.

12. Under a subsection “Appropriate health care seeking behavior” several studies include obtaining care from pharmacies as appropriate health care seeking behaviour (HSB), how come you are doing this differently?

**Author’s response**: We also took references of several studies, obtaining medicines from pharmacies without prescription/doctor’s consultation is considered as in-appropriate health care seeking behavior. Obtaining medicines from pharmacies without any consultation comes under self- medication, because of this irrational use of medication; antibiotic/drug resistance is a big issue in Nepal, We had taken the reference of studies from Yemen, India and North- West Ethiopia. Full citations of these studies are mentioned below.

Kalita D, Borah M, Kakati R, Borah H. Primary Caregivers Health Seeking Behaviour for Under-Five Children : A Study in a Rural Block of Assam, India. Ntl J Community Med. 2016;7(11):868–72.

Webair HH, Bin-Gouth AS. Factors affecting health seeking behavior for common childhood illnesses in Yemen. Patient Prefer Adherence. 2013;7:1129–38.

Molla Simieneh M, Mengistu Y, Gelagay AA, Gebeyehu MT. Mothers’ health care seeking behavior and associated factors for common childhood illnesses, Northwest Ethiopia: community based cross-sectional study.

13. Can you refer to “Ng'ambi W, Mangal T, Phillips A, Colbourn T, Mfutso-Bengo J, Revill P, Hallett TB. Factors associated with healthcare seeking behaviour for children in Malawi: 2016. Trop Med Int Health. 2020 Dec;25(12):1486-1495. doi: 10.1111/tmi.13499. Epub 2020 Oct 19. PMID: 32981174.” for some operational definition of HSB.

**Author’s response**: As we had conducted this study on 2018, we had defined operational definitions ourselves before the conduction of study taking reference of relevant literature, now study have already conducted, we can’t modify our operational definitions. Operational definitions are always defined before the conduction of study, not after the completion of study, as operational definitions involves variables; these are set before data collection.

Results
14. Format the tables for characteristics to look like this.
Patient characteristics n (%)
Total 255229 (100.0)
Gender
Male 122610 (48.0)
Female 125275 (49.1)
Missing 7344 (2.9)
Location
Rural 168258 (65.9)
Urban 86971 (34.1)
Age at Sample draw (in months)
0-1 145622 (57.1)
2-5 74707 (29.3)
[6-11 21307](tel:6-11%2021307) (8.4)
[12-17 3337](tel:12-17%203337) (1.3)
[18-24 1902](tel:18-24%201902) (0.8)
Missing 8354 (3.3)
Region
Northern 22897 (9.0)
Central 72,633 (28.5)
Southern 159699 (62.6)
Year Sample drawn
[2013 16308](tel:2013%2016308) (6.4)
[2014 25858](tel:2014%2025858) (10.1)
[2015 41271](tel:2015%2041271) (16.2)
[2016 41178](tel:2016%2041178) (16.1)
[2017 42252](tel:2017%2042252) (16.6)
[2018 43370](tel:2018%2043370) (17.0)
[2019 36372](tel:2019%2036372) (14.3)
2020* 7741 (3.0)
Missing 879 (0.3)

15. In all tables do not combine the mean or SD if the headings are number and percentage

**Author’s response**: Thank you for your feedback, I have updated the tables accordingly.

16. All tables should not have lines crossing them

**Author’s response**: Thank you for your feedback, I have updated the table accordingly.

17. Include the illnesses (like fever, diarrheas) as appriori variables in the final multivariate model as these form key part of your analysis. Also include age and sex of the child as appriori variables.

18. We are not sure of the P-value for Table 5. Is it for crude or adjusted estimates? I would format the table to look like:-
Characteristics (n=26386) Bivariate analysis Multivariate analysis
OR (95%CI) P-value OR (95%CI) P-value
Age group
15-19 1.00 1.00
20-24 1.10 (1.00-1.21) 0.06 1.26 (1.13-1.41) <0.001
25-29 1.07 (0.96-1.18) 0.22 1.42 (1.24-1.62) <0.001
30-34 1.06 (0.95-1.18) 0.33 1.48 (1.27-1.72) <0.001
35-39 1.07 (0.95-1.21) 0.27 1.61 (1.36-1.92) <0.001
40-44 0.91 (0.78-1.07) 0.25 1.47 (1.19-1.81) <0.001
45-49 1.02 (0.80-1.30) 0.88 1.77 (1.33-2.34) <0.001
Year
2004/5 1.00 1.00
2010 1.23 (1.14-1.32) <0.001 1.21 (1.12-1.31) <0.001
2015/16 2.19 (2.03-2.37) <0.001 2.12 (1.97-2.29) <0.001
Region
North 1.00
Centre 1.03 (0.94-1.12) 0.55
South 0.96 (0.88-1.05) 0.40
Number of previous children ever born
1 1.00 1.00
2-3 0.79 (0.74-0.85) <0.001 0.70 (0.64-0.76) <0.001
4-5 0.74 (0.68-0.80) <0.001 0.62 (0.55-0.70) <0.001
6+ 0.69 (0.63-0.75) <0.001 0.59 (0.51-0.69) <0.001
Education level
None 1.00 1.00
Primary 1.22 (1.13-1.32) <0.001 1.09 (1.00-1.18) 0.05
Secondary 1.69 (1.54-1.86) <0.000 1.24 (1.11-1.39) <0.001
Tertiary 4.36 (3.42-5.57) <0.001 2.35 (1.80-3.06) <0.001
Wealth index quintile
Poorest 1.00 1.00
Poorer 1.07 (0.99-1.16) 0.10 1.07 (0.99-1.17) 0.09
Middle 1.09 (1.00-1.18) 0.05 1.10 (1.01-1.20) 0.024
Richer 1.17 (1.07-1.28) <0.001 1.15 (1.05-1.26) 0.002
Richest 1.47 (1.35-1.60) <0.001 1.23 (1.11-1.36) <0.001
Residence
Urban 1.00
Rural 0.78 (0.72-0.84) <0.001
Sources of antenatal care knowledge
Frequency of listening to radio
Less than once a week 1.00
At least once a week 1.00 (0.95-1.05) 0.97
Frequency of watching television
Less than once a week 1.00 1.00
At least once a week 1.44 (1.31-1.58) <0.001 1.13 (1.02-1.26) 0.025
Barriers to access antenatal care
Permission to visit health services
No problem 1.00
Big problem 1.10 (1.02-1.19) 0.01
Money to pay for health services
No problem 1.00
Big problem 0.88 (0.83-0.92) <0.001
Distance to health facilities
No problem 1.00
Big problem 0.93 (0.88-0.98) 0.005
Presence of companion
No problem 1.00
Big problem 0.98 (0.92-1.04) 0.50
No drugs at health facility
No problem 1.00
Big problem 1.28 (1.21-1.35) <0.001
No female provider
No problem 1.00
Big problem 1.01 (0.95-1.09) 0.64
Marital status
Never married 1.00 1.00
Married 1.20 (1.02-1.41) 0.026 1.65 (1.39-1.96) <0.001
Widowed 1.01 (0.78-1.34) 0.89 1.50 (1.13-1.99) 0.005
Divorced 1.08 (0.90-1.29) 0.40 1.50 (1.24-1.81) <0.001

**Author’s response:** It is not crude, it is adjusted estimates.

Discussion
19. Start the discussion with a summary of the key results of your study and then move on to discuss them in light of other relevant literature.
20. The findings from this paper may be helpful to your discussion and conclusion. Consider citing it as well.
Ng'ambi W, Mangal T, Phillips A, Colbourn T, Mfutso-Bengo J, Revill P, Hallett TB. Factors associated with healthcare seeking behaviour for children in Malawi: 2016. Trop Med Int Health. 2020 Dec;25(12):1486-1495. doi: 10.1111/tmi.13499. Epub 2020 Oct 19. PMID: 32981174.
21. Don’t repeat results in the discussion but rather compare your key results with those of other studies and explain any disparity or agreement.
Conclusion
22. This section should provide policy insights as well as alignment of the study findings with the SDGs as well as universal health coverage.
23. Include the policy relevance of your study findings as well.

**Author’s response:** We have included policy relevance of our findings in the conclusion section.
